# The Relationship Between Dementia Severity & Caregiver Preferences for Decision Making Role Regarding Mammography

**DOI:** 10.1093/geroni/igab046.3537

**Published:** 2021-12-17

**Authors:** Molly Frank, Seho Park, Kathleen Lane, Alexia Torke, Mara Schonberg, Greg Sachs, Peter Schwartz, Nicole Fowler

**Affiliations:** 1 Indiana University School of Medicine, IU Center for Aging Research, Indianapolis, Indiana, United States; 2 Department of Biostatistics, School of Medicine, Department of Biostatistics, School of Medicine, Indiana, United States; 3 Indiana University School of Medicine, Indianapolis, Indiana, United States; 4 Beth Israel Deaconess Medical Center, Boston, Massachusetts, United States; 5 Center for Bioethics, Indianapolis, Indiana, United States

## Abstract

The incidence of Alzheimer’s disease and related dementias (ADRD) and breast cancer increases with age. Despite being one of the most effective ways to diagnose breast cancer early, mammography in ADRD patients comes with an increased risk of treatment complications and false-positive results. Family caregivers are often involved in the decision-making process, and this study evaluates the relationship between dementia severity and caregiver preferences when making decisions about mammography with the patient alone, and with the patient and doctor. We included 181 caregivers from the Decisions about Cancer screening in Alzheimer’s Disease trial, which uses the Dementia Severity Rating Scale (DSRS) to assess dementia severity and a modified Control Preferences Scale (CPS) to assess each caregiver’s preferred decision-making approach. Multinomial logistic regression models evaluated the relationship between DSRS and CPS categories (active, passive, and collaborative), while controlling for the caregivers’ age, sex, race, education, and relationship to patient. Model 1 examined the caregivers’ preferred role with the patient, and it found a significant association between increased dementia severity and preference for a collaborative approach (p<0.001) or passive approach (p<0.05) compared to an active approach. Model 2 did not find a significant association between dementia severity and the caregivers’ preferred role when making decisions with the patient and doctor; however, those with increased age and education were more likely to prefer an active role. The association between dementia severity, caregiver characteristics, and decision-making preferences supports the need for approaches to support ADRD caregivers with medical decision making.

